# Application of Protein Toxins as Cell Biological and Pharmacological Tools

**DOI:** 10.3390/toxins14040242

**Published:** 2022-03-28

**Authors:** Ludger Johannes

**Affiliations:** Institut Curie, Université PSL, U1143 INSERM, UMR3666 CNRS, Cellular and Chemical Biology Unit, 26 Rue d’Ulm, CEDEX 05, 75248 Paris, France; ludger.johannes@curie.fr

Protein toxins from bacteria and plants are a serious threat to human and animal health. However, because of their intimate interactions with host cells, they have also become valuable tools to molecularly dissect cell biological functions that range from endocytosis and intracellular trafficking to cell signaling and apoptosis. Several characteristics such as ease for biochemical handling and the robustness of phenotypes are responsible for their success as cell biological tools. The in-depth understanding of their activities has, in turn, also attracted attention to their use as pharmacological tools to manipulate cellular processes that misfunction in disease situations or that can be targeted for therapeutic intervention. Cancer immunotherapy by immunotoxins or toxin subunit-based vaccines are only some of the many possibilities for using toxins in biomedical research.

In this Special Issue of *Toxins*, primary research papers and review articles have been assembled that address the aspects that are summarized above. The reader is thereby provided with an up-to-date perspective on some of the most recent and dynamic contributions of toxins to fundamental cell biology and applied cellular pharmacology research.

Siukstaite and colleagues have designed a bispecific chimeric lectin, termed Janus lectin [1]. Because of its oligomeric nature, apparent affinities in the nanomolar range were reached for sialic acid and fucose. The authors demonstrate that by bridging glycans on liposomes and on cells, the Janus lectin drives the uptake of the former into the latter. Such engineered lectin may therefore be exploited for the targeting of liposome-encapsulated drugs to tumors that are often characterized by hypersialylation.

Keshtvarz and colleagues have engineered cytolethal distending toxin (CDT) to reduce its immunogenicity, thereby making it more attractive as an agent for targeted therapy by exploiting its nuclease activity [2]. B cell epitopes were identified using computational methods, and their mutation was indeed found to decrease immunogenicity and to increase stability of CDT, making it an attractive candidate for the development of immunotoxin strategies.

Selyunin and colleagues have observed that the chemotherapeutic agent tamoxifen protects cells against Shiga toxins 1 and 2 by inhibiting their intracellular trafficking from early endosomes to the Golgi apparatus [3]. Initially, the authors thought that this effect was due to a modification of endosomal pH by tamoxifen. They have then accumulated evidence in favor of an effect of tamoxifen on the retromer-dependent formation of tubular trafficking intermediates between early endosomes and the Golgi apparatus.

Detzner and colleagues have worked on primary human renal cortical epithelial cells (pHRCEpiCs) to analyze the distribution of different molecular species of the Shiga toxin receptor, the glycosphingolipid Gb3, in density gradient fractions that were obtained after detergent solubilization of cell membranes [4]. As expected, Gb3 species with saturated acyl chains were preferentially found in detergent resistant membranes (DRMs), while those with unsaturated acyl chains were mostly in non-DRMs. Despite a large overall bias of Gb3 for DRMs in these cells, toxicity assays revealed that they were only moderately sensitive to the Shiga-like toxins Stx1a and Stx2a, suggesting that other factors also contribute to their intoxication.

Tartour and Johannes have reviewed the use of the receptor-binding B-subunit of Shiga toxin, termed STxB, as an antigen delivery tool for mucosal vaccination [5]. The vectorization of viral or tumor antigens by STxB indeed leads to the induction of secretory IgAs and of tissue resident memory T cells, which are the types of immune responses for which efficacity has been demonstrated in viral infection models and against mucosal tumors of the head and neck. In this review, STxB-based vaccines are extensively compared to other vaccination approaches, and it is pointed out how combinations with other treatment modalities such as immune checkpoint inhibitors can lead to therapeutically beneficial synergies.

Robert and Wiels have reviewed the use of Shiga toxin as an antitumor tool with an emphasis on the direct targeting of tumor cells by exploiting an overexpression of the Shiga toxin receptor, the glycosphingolipid Gb3 [6]. A focus was put on Burkitt lymphoma, on which historically the first anti-Gb3 antibody was generated and shown to have the capacity to induce their apoptosis. The authors discuss the different Shiga toxin-based modalities that were developed for the imaging and the treatment of tumors.

Kenworthy and colleagues have reviewed the literature on the use of cholera toxin as a tool in cell biology and biophysics research [7]. Cholera toxin has been shown to have phase active properties in membranes that result from its oligomeric nature and the capacity to reorganize one of its main receptor molecules, i.e., the ganglioside GM1. Cholera toxin can also reshape membranes to which it is bound in relation to its endocytic entry into cells. The authors point out the existence of high similarity between cholera toxin and the members of the Shiga toxin family in what concerns these membrane reshaping activities.

Pezeshkian and colleagues have reviewed computational approaches to study the entry of Shiga and cholera toxins into host cells [8]. The authors illustrate how simulations have allowed the development of novel hypotheses on mechanisms by which toxin-driven reorganization of membrane lipids leads to defined biological outcomes. Notably, how toxin molecules are clustered on target cell membranes and membrane curvature is generated for the formation of tubular endocytic pits according to the glycolipid-lectin (GL-Lect) hypothesis has been explored by these simulation techniques.

Lingwood has reviewed the therapeutic uses of Shiga toxin and its subunits [9]. A specific focus is laid on the holotoxin itself and the possibility to target and eliminate Gb3 expressing tumors. Furthermore, it is also reviewed how the toxin’s capacity to interact with the ER-associated degradation machinery (ERAD) allows rescuing the exit from the ER of proteins that are misfolded and involved in pathologies such as cystic fibrosis and Gaucher disease.

Sandvig and colleagues have reviewed how protein toxins, and notably the plant toxin ricin and the bacterial Shiga toxin, have been exploited as tools in cell biology research [10]. The focus of this review is on the exploration of the role of defined lipid classes and species in intracellular transport, which is an aspect that has been made better accessible through advances in mass spectrometry. It is pointed out how the expression of different lipid classes is correlated, and how compensation is observed under certain conditions.

## Data Availability

Not applicable.
